# Autonomous schema markups based on intelligent computing for search engine optimization

**DOI:** 10.7717/peerj-cs.1163

**Published:** 2022-12-08

**Authors:** Burhan Ud Din Abbasi, Iram Fatima, Hamid Mukhtar, Sharifullah Khan, Abdulaziz Alhumam, Hafiz Farooq Ahmad

**Affiliations:** 1 Department of Computer Science, Bahria University, Islamabad, Pakistan; 2 Schema App-Hunch Manifest Inc, Guelph, Canada; 3 Department of Computer Science, College of Engineering and Physical Sciences (EPS), University of Birmingham Dubai, Dubai, United Arab Emirates; 4 PAF-Institute of Applied Sciences and Technology, Haripur, Pakistan; 5 Computer Science Department, College of Computer Sciences and Information Technology (CCSIT), King Faisal University, Al-Ahsa, Saudi Arabia

**Keywords:** Schema.org, Search engine optimization, Semantic search, Unstructured data, Content discovery

## Abstract

With advances in artificial intelligence and semantic technology, search engines are integrating semantics to address complex search queries to improve the results. This requires identification of well-known concepts or entities and their relationship from web page contents. But the increase in complex unstructured data on web pages has made the task of concept identification overly complex. Existing research focuses on entity recognition from the perspective of linguistic structures such as complete sentences and paragraphs, whereas a huge part of the data on web pages exists as unstructured text fragments enclosed in HTML tags. Ontologies provide schemas to structure the data on the web. However, including them in the web pages requires additional resources and expertise from organizations or webmasters and thus becoming a major hindrance in their large-scale adoption. We propose an approach for autonomous identification of entities from short text present in web pages to populate semantic models based on a specific ontology model. The proposed approach has been applied to a public dataset containing academic web pages. We employ a long short-term memory (LSTM) deep learning network and the random forest machine learning algorithm to predict entities. The proposed methodology gives an overall accuracy of 0.94 on the test dataset, indicating a potential for automated prediction even in the case of a limited number of training samples for various entities, thus, significantly reducing the required manual workload in practical applications.

## Introduction

The rise of the Internet and the rapid growth in web services has reshaped the process of information provision and access on the web. The idea that information is the key to economic and intellectual growth has been wholeheartedly adopted in the rapidly evolving digital world. Organizations maintain content-rich websites to cater to the informational needs of the users. Filtering millions of web pages to identify pieces of information that are of interest leads to an improved user experience across different domains such as question answering (Vanessa et al., 2011), information visualization (Ansari, Barati & Martin, 2022), information integration (Ankolekar et al., 2007), and personalized and context-specific information (Şah et al., 2007).

To bridge the gap between information on the Internet and end users’ needs, search engines have emerged as major sources of content discovery and provision to an extremely large number of online users. It is a common practice for internet users to use search engines as a starting point in their quest to fulfill information requirements. Information and content providers employ additional means to influence the results shown by search engines such as online advertisements and optimization of website content through a well-known process search engine optimization (SEO) (Gudivada, Rao & Paris, 2015). SEO has a significant impact on the ranking of content and websites in search engine results, by improving awareness among users for specific content, and even greatly enhancing the market share for the products (Bhandari & Bansal, 2018; Lewandowski, Sunkler & Yagci, 2021; Akram et al., 2010). Based on the huge role SEO plays in today’s digital world, SEO has evolved to become a multi-billion-dollar industry (Schultheiß & Lewandowski, 2020).

A search engine discovers information on the World Wide Web (WWW) and adds its references to its database making it searchable to users through a process known as indexing. Once a website has been indexed, the search engine keeps checking back routinely to see if there have been any changes and updates; in this way, search engines keep the latest information that can be served to the users upon request. Search engines have evolved to handle different forms of data as well as different uses such as text, images, and video games. It is estimated that more than 80% of the data on the web is unstructured (Bhatia, 2019) and search engines are evolving to index and extract useful information from this data for answering user queries. They employ various techniques for information storage, processing, ranking, and retrieval for filtering the most relevant results (Bevendorff et al., 2018; Jing et al., 2021; Zhang et al., 2018).

Despite the technological progress made by search engines, there is a gap between how the content on the web is developed and how the search engines process it to answer user queries. Slow adoption of semantics technologies by the content creators may be attributed to a lack of appropriate methods and human resources among other factors. Due to this, a big portion of the web content remains unprocessed by the search engines for extraction of semantic information. Creating value from this data requires innovative and easy-to-use semantic technology (Kim et al., 2018) This article addresses the gap by structuring the contents on the Web pages using semantic technology.

In the next subsection, we discuss the evolution of search engine optimization (SEO) techniques and the need for structured information. In addition, we present an overview of the recent developments in natural language processing (NLP) that lead to the enhancement of semantic search.

### Emergence of SEO

In the presence of digital media, organizations have become more conscious of their information on product quality, brand identity, and user engagement through online and offline media. Organizations adopt a proactive approach to build the visibility of products, services, and initiatives. Professionals working in this domain are identified as digital marketers, webmasters, or SEO specialists. We collectively refer to these roles as “SEO professionals” in the latter text. A key responsibility for these roles is to have a deep understanding of how search engines work, monitor website traffic, and use strategies to improve ranking in search results. The goal is to increase visibility and website traffic as well as to increase engagement of users visiting the website, thus, focusing on quantity and quality at the same time (Aryshandy et al., 2021).

Search engines do not reveal the complete criteria and weightage of factors influencing SEO for claiming impartial services to the users as well as fair play among business competitors (Schultheiß & Lewandowski, 2020). But they share a set of general guidelines and recommend best practices to be followed. SEO professionals follow these guidelines rigorously in the pursuit of ranking their web pages among the top search results. Due to the lack of any semantics and semantic structures imposed by the Hypertext Markup Language (HTML), many of the initial SEO techniques were based on syntactic structuring and placement of keywords on a web page. As the professionals learned and uncovered the SEO techniques used by different search engines, it became evident that many of such techniques could be abused (Dye, 2008).

### The need for structured information

The necessity to identify useful information and its potential to transform the digital arena has led to the idea of representing data in such a way that it becomes understandable to machines as well. Unfortunately, HTML, the language for content presentation of web pages on the WWW was designed without any focus on data semantics. While the HTML “tags” could accomplish the task of structuring the content across a web page, from an information retrieval point of view, these tags did not convey any semantics that could be used for proper indexing of structured information. This “unstructured” data on web pages posed a big challenge for appropriate data indexing and, thus, successful handling of the complicated user queries by search engines. Semantic elements were introduced in HTML5 to facilitate the effective searching of web page content (Anthes, 2012). Use of elements such as <nav>, <article>, <section>, <aside>, and <address> provide additional information to the search engines about the context of the content, therefore enabling the search engines to find content that may be relevant to search terms used by the Internet users. However, the use of semantic elements alone could not solve the problem of identification of entities and their relationships.

### The advent of the semantic web

The notion of semantic web is enabled by providing machine-readable descriptions of the content available on the web (Berners-Lee, Hendler & Lassila, 2001). In line with the goals of SEO, search engines try to better serve their users through the tools and technologies related to the semantic web. The notion of the semantic web is realized using web ontologies. An ontology formalizes the way a certain concept may be referred to and defined and how this concept is related to other concepts (Khadir, Aliane & Guessoum, 2021). Web ontologies are generally composed of a taxonomy and a set of inference rules. The taxonomy dictates the rules to define different classes of objects and the relationship between the classes, which may be used to describe the content of a web page also referred to as metadata. The use of metadata is a common practice to inform search engines about the nature of content present on a website (Baye, Santos & Wildenbeest, 2016).

### Development of the schema.org vocabulary

*Schema.org* (https://schema.org/) provides a mechanism to structure the information present on web pages and make it more discoverable for search engines. It was a joint initiative by multiple leading search engines such as Google, Microsoft, Yahoo, and Yandex. It provides a collection of shared vocabularies that can be used by webmasters to mark up their pages. The vocabulary of *Schema.org* is organized as a collection of schemas that can be used to mark up a webpage in ways that can be understood by the major search engines to facilitate semantic search (Guha, Brickley & Macbeth, 2016; Patel-Schneider, 2014). Schemas are composed of “types” or “classes”, where each type is defined in terms of a set of “properties”.

Classes in schema.org are used to identify broader categories of objects and concepts such as a person, organization, event, and products (Guha, Brickley & Macbeth, 2016). Like the real-world, each class has some properties, values of those properties distinguish different objects of the same class. Objects of different or same classes may also be related through these properties. For example, a *Person* may have an occupation and be affiliated with one or more organizations. An object of *Person* may have children who will again be represented by the class Person. If a person is a faculty member at a certain institute, his/her information may be defined in terms of the properties from the classes Person, Organization, and Occupation. Properties such as “name”, “image”, “email”, “address”, “honorificPrefix” in the Person class and “name”, “URL”, “department” in the Organization class could be utilized to structure information related to that specific faculty member. For example, by using Schema.org vocabulary to define the contents of our webpage, we can enable a search engine to filter the results more accurately for user searches such as “Who is the Head of Department (HOD) of Computer Science at Melbourne university” or “What is the email address of HOD of Computer Science at Chicago university”. Similarly, newly added information such as the contact number or email address of the HOD will be readily available for user queries.

### Advances in natural language processing

**Figure 1:**
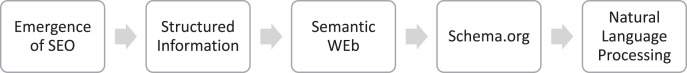
Stages of development leading to semantic search.

With the popularity of deep neural networks, researchers found their applications in the field of natural language processing (NLP). In the latest race of SEO, search engine giants like Baidu, Bing, Google, and Yandex are striving for achieving a near-human level of understanding of user’s search queries. These search engines can now understand question and answer, identify missing information, summarize long articles, and recommend related content to the users. The underlying technology behind these search engines is language models that use word embeddings based on lexical semantics (Wang, Zhou & Jiang, 2020). By applying NLP, search engines can identify entities and their relationships from the text. Figure 1 summarizes the stages of development of technologies, leading to the current state of Semantic search (Hitzler, 2021).

Identifying entities from the text is referred to as Named Entity Recognition (NER) and it has been a focus of research for over three decades (Manning & Schutze, 1999). While researchers transitioned from rule-based approaches to machine learning models, the topic remains an intriguing research problem (Nadeau & Sekine, 2007). There has been significant progress in recognizing some of the common entity types from long text and paragraphs (Yadav & Bethard, 2019). Research shows that different types of named entities such as person names, food names, and location names can be identified using distant learning (Chou & Chang, 2017; Chou et al., 2020).

### Challenges in using NLP for semantic modelling

In the previous sub-section, we discussed that the advances in deep learning and NLP-based models have led to an unprecedented level of sophistication in understanding user queries. On the upside, all these developments are related to search engine indexing and content processing and no changes have been applied to the content available on the web (Saini et al., 2022; Izo, Oliveira & Badue, 2022). In other words, while the decades-old web pages remain untouched on the web servers, it is only the search engine's algorithms that have been updated to perform improved analysis on the indexed content. On the downside, however, there are some limitations of the NLP-based models.

First, the language models rely on complete language structures like sentences, paragraphs, and articles for the identification of semantic concepts to identify entities and their relationships. Therefore, in principle, these models work very well on content like news, blogs, and social media posts that contain complete language units; but they fail to function in situations when the content is made up of keywords or short phrases instead of complete sentences (Khattak et al., 2019). Since a large part of the web content is present in online directories, e-commerce, and trade websites, institutional pages, art, and technical portfolios, and medical and law cases, all of which may contain important concepts and entities in the form of keywords and phrases, the NLP-based models are unable to extract information from these web pages. This poses a significant limitation in the development and adoption of technologies related to semantic modeling.

Second, while the language models are good at capturing web content in its most generic form, they are unable to identify the subtleties attached to a particular domain. For example, in the academic domain, we have concepts such as teacher, instructor, lecturer, professor, chairman, faculty member, *etc*. A person may have one of these roles at a time, *e.g*., an instructor; or may occupy multiple roles at the same time (professor, chairman, and faculty member). The specificity of these concepts poses a challenge that is yet to be tackled by the language models (Li et al., 2020).

Thus, the unstructured text which is not in the form of sentences and paragraphs may hold the information required by search engine users, as per the examples already discussed. A possible solution is to use human intervention for updating the content. Given the amount of information available on the web, this approach seems unreasonable. As the research on automated extraction of information from such content through NLP techniques is limited but holds the potential for a huge impact, we propose the use of an automated procedure for entity and relationship extraction from web pages.

### Research contribution

This research addresses the challenge of the need for human intervention to populate schema markups with the correct information. Automation of the process using the proposed approach can facilitate the adoption of technologies associated with the Semantic Web and SEO. The approach can significantly reduce the effort required for the enrichment of semantic models. We propose to build such models based on the existing standard of JavaScript Object Notation-Linked Data (JSON-LD), a widely accepted way of representing semantic data present on the web. Our approach is generic; however, for evaluating the approach on web pages of a particular domain, we use a directory of web pages of academic websites as a case study. Instead of developing a custom ontology or vocabulary, our approach benefits from the use of existing ontology from Schema.org for concept mapping. We can summarize our contributions as follows:

1. We propose an automated approach to infer the type of entity for short texts that can be mapped to its associated markups from Schema.org vocabulary.
2. We employ random forest and LSTM to show the potential of both machine learning and deep learning techniques in tackling the underlying challenge of data scarcity. The methodology is designed considering the natural difference in the frequency of occurrence of entities on web pages of a given domain.
3. We show that the approach is effective for web pages for the academic domain covering a range of entities such as person, department, educational qualification, and ranks, with the potential to be adopted for the web content of any given niche such as health and e-commerce.

The rest of the article is organized as follows:

“Literature Review” dives into the literature to present a deeper understanding of related scientific works, “Methodology” explains the methodology applied in this research from data preparation to model building and implementation. “Experimental Evaluation and Discussion” describes the experiments performed using the proposed mode and their performance evaluation on a public dataset. “Conclusion” concludes this research article.

## Literature review

Autonomous knowledge extraction from diverse semi-structured and unstructured web pages to facilitate SEO is an important challenge. Limited support for the types of objects by leading search engines and service providers points to the major gap in the domains of SEO and the semantic web. Although a well-researched user interface design helps to make the task relatively less hectic, such solutions have a long way to go in terms of scalability, and the need for an autonomous solution is evident. With the availability of a reliable knowledge extraction and labeling (entity recognition) mechanism, an automated system to populate semantic models based on the ontology structure described by Schema.org can be designed and used to facilitate semantic search features for web content visibility in search engine optimization. Proposing a solution for such a broad problem can be a challenging task, while our experiments narrow down to a specific domain to show the effectiveness of our approach the research focuses on designing a solution that can be generalized to different domains.

### Related research in semantic web and ontologies

Semantic web and ontology modeling have been a focus of research for several years. Research has targeted many different aspects to achieve the goal of structuring the information present on the Internet. Since the outcome of ontology modeling techniques is strongly dependent on the nature of the data itself (Saeeda et al., 2020), researchers have proposed specialized ontologies to better describe various aspects of a domain. As an example, Stolz, Hepp & Hemminger (2017) proposed a vocabulary for the domain of garments that can be used to express web page content and enable the Semantic web. However, such efforts remain limited in creating a large-scale impact unless officially adopted by Schema.org as a standard.

Web pages may contain a large amount of data with variations in the type of information presented on a single page. While schema.org provides a taxonomic scheme to be followed, information mapping still needs to be managed manually. This requires human effort and becomes a bottleneck when it comes to scaling content-heavy web pages. An additional factor for consideration is the domain knowledge of individuals handling the task of labeling. In most cases, an individual with domain knowledge and expertise would be considered more appropriate than a layman. Even if the availability of skilled human resources were not a limiting factor, the use of human-labeled data is still not guaranteed to yield optimal labeling; research shows that HTML block labeling can be a tricky task, where the same block may end up being labeled differently by different people (Griazev & Ramanauskaitė, 2021).

Nie et al. (2020) proposed using semantic augmentation based on similar words from pre-trained embeddings. In their work, they used benchmark datasets from two different languages and employed pre-trained embeddings for each of those languages. While the proposed approach performed better than the baseline defined by the authors, the use of semantic augmentation for each token in the sentence renders the approach unsuitable in the context of our research. In separate research on named entity recognition, social media posts containing text as well as images were used. While the approach yielded improved performance, the availability of visual data along with every piece of text is not practical for every webpage. Moreover, researchers filtered out short tweets containing less than three words from the dataset; therefore, the results are only representative of model performance on long text from social media posts (Asgari-Chenaghlu et al., 2022). In another research multi-modal based named entity recognition technique has been proposed for short text. The research, however, only differentiates between common entities such as a person, location, organization, and miscellaneous (Moon, Neves & Carvalho, 2018). Fu, Huang & Liu (2021) propose an interesting approach to tackle entity recognition, using spans of texts to label a sequence of tokens. The approach yielded promising results for the datasets, where each of them mainly consisted of long texts such as news articles and telephone conversations.

Recently researchers are turning towards more challenging types of data sources for semantic analysis and investigating scenarios such as short-text generated on social media where little or no surrounding context is available (Moon, Neves & Carvalho, 2018; Nie et al., 2020). While deep learning algorithms have proven their effectiveness in these tasks, it may be noted that most research on NER focus on a select few entities such as location, person, organization, and miscellaneous (Nguyen, Nguyen & Rao, 2020). To extend this concept to more entities, research has been done in the domain of agriculture (Guo et al., 2022), legal documents (Leitner, Rehm & Moreno-Schneider, 2019) and the fashion industry (Chilet, Chen & Lin, 2016), *etc*. De Freitas Veneroso & Ribeiro-Neto (2018) utilized deep learning to identify faculty names from directories of webpages maintained on the websites of academic institutes. The work found the traditional approaches for NER to be less effective as compared to deep learning algorithms in persons’ name extraction from web pages. An obvious limitation of this research was that it only covered one type of entity, such as a person's name.

In the context of information extraction from web pages, traditional approaches require the knowledge of the Document Object Model (DOM) which is a memory-based representation of a web page for manipulation through programming languages, such as JavaScript, Java, or Python, *etc*. The need for foreknowledge of DOM structure renders the approaches ineffective for unseen web pages even when the nature of the content is known to be the same. Researchers have attempted to tackle this scenario for the identification of numerical data from web pages using summarization and NER (Bhardwaj et al., 2021). The results for NER were found to be promising; however, a key limitation of this approach was that it focused only on the identification of numerical data. Inspired by this work, we performed prediction on our dataset using Spacy (https://spacy.io/), a widely used library by the natural language research and development community. It has a built-in module for named entity recognition that supports the prediction of many different entities such as people, dates, events, buildings, countries, languages, locations, currencies, organizations, *etc*. It may be noted that entities supported by Spacy are generalized to support a wide range of natural language texts. The performance on our dataset was insignificant, therefore limiting us to form a baseline. We speculate that the reasons may be multi-folds, the most important one being the fact that the content of our dataset is not in the form of grammatical structures such as sentences and paragraphs. On the other hand, the entities in this research cover a specific kind of detail that can be utilized to populate the semantic blocks of linked data. Another key difference is that the entities in our research are related to form an ontology which can be expected since Schema.org is designed to cater to a broad range of topics and objects that may be interrelated.

### Tools for search engine optimization (SEO)

Various tools and techniques are being developed to facilitate search engine optimization. These tools and services may be offered by search engine service providers or by third-party service providers. Google Search Console (https://search.google.com/search-console/about) is a service offered by the Google search engine to monitor websites and analyze traffic. It allows SEO professionals to identify the key search terms that generate traffic, the most visited pages as well as the broken links on a website. As a result, they can better understand the search terms and optimize the content of a website to improve the visibility and ranking in search results.

SEO professionals use several techniques ranging from the use of security certificates to on-page modifications leading to improved ranking in search engine results (Akram et al., 2010). They describe the contents of a web page using appropriate Schema.org classes for each piece of information that needs to be represented. Depending on the availability of data we can populate relevant properties, thus, making it easier for search engines to discover the content relevant to a user’s query. The use of appropriate tags as per the rules of a certain ontology and accurately populating them for each web page is important in the context of SEO.

**Figure 2:**
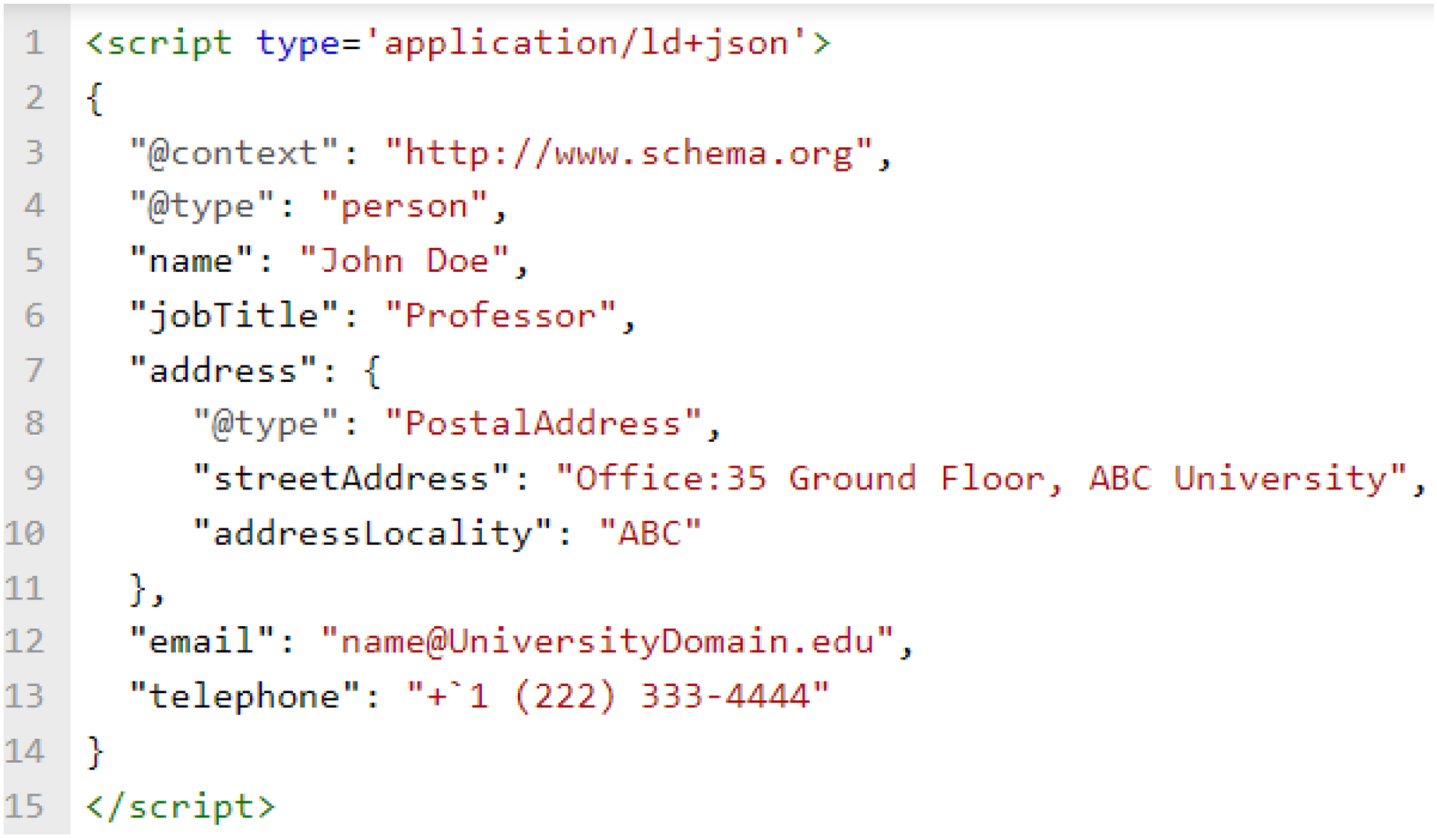
JSON-LD block of professor’s information.

JavaScript Object Notation for Linked Data (JSON-LD) is the W3C standard recommended by Schema.org to represent semantic information for the web (Sporny, Kellogg & Lanthaler, 2014; Kellogg, Champin & Longley, 2020). After entities are identified from the webpage, software programs can use the identified property values to enrich pre-defined JSON-LD blocks. As an example, we show a JSON-LD block in Fig. 2 which represents a person’s details. While not the focus of current work, we hope to see further contributions by researchers in this area to make the task of JSON-LD block creation and enrichment easier for organizations.

**Figure 3:**
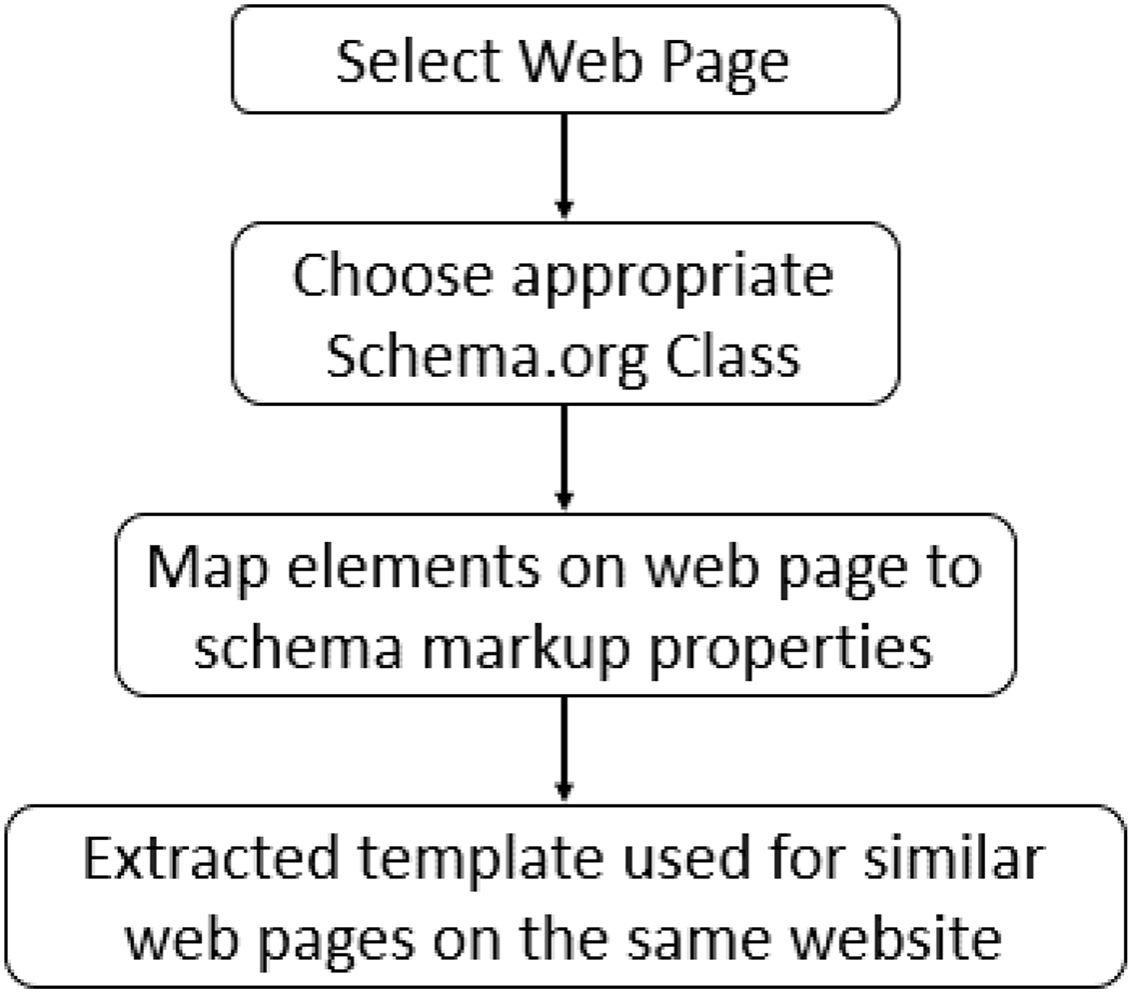
Workflow of existing tools.

Existing methods to create a mapping between the web page content and Schema.org tags involve plugins and interactive tools that require human users’ intervention. Google has deployed its version of a markup tool to support webmasters in generating JSON-LD blocks from the web page known as “Structured Data Markup Helper” (https://www.google.com/webmasters/markup-helper). At the time of writing, it only supports nine types of objects to generate appropriate JSON-LD blocks. These types include articles, events, local businesses, restaurants, book reviews, films, products, software applications, data sets, job postings, question answers pages, and TV episodes. There is no obvious reason for how these types were chosen and why not the others; for instance, the Person type can be very useful for indexing information related to individuals working across various domains. This indexed information may then be found by users who are looking for a specific individual based on their names, domains, or affiliations. As an example, information about faculty members at an academic organization may be of interest to other academicians. This scenario holds valid for patients looking for a doctor, and students looking for a professor or manager in search of a skillful employee. Several other organizations (https://www.schemaapp.com/products/schema-app-highlighter/) (https://hallanalysis.com/json-ld-generator/) working in this domain offer similar solutions with a generalized workflow shown in Fig. 3.

**Figure 4:**
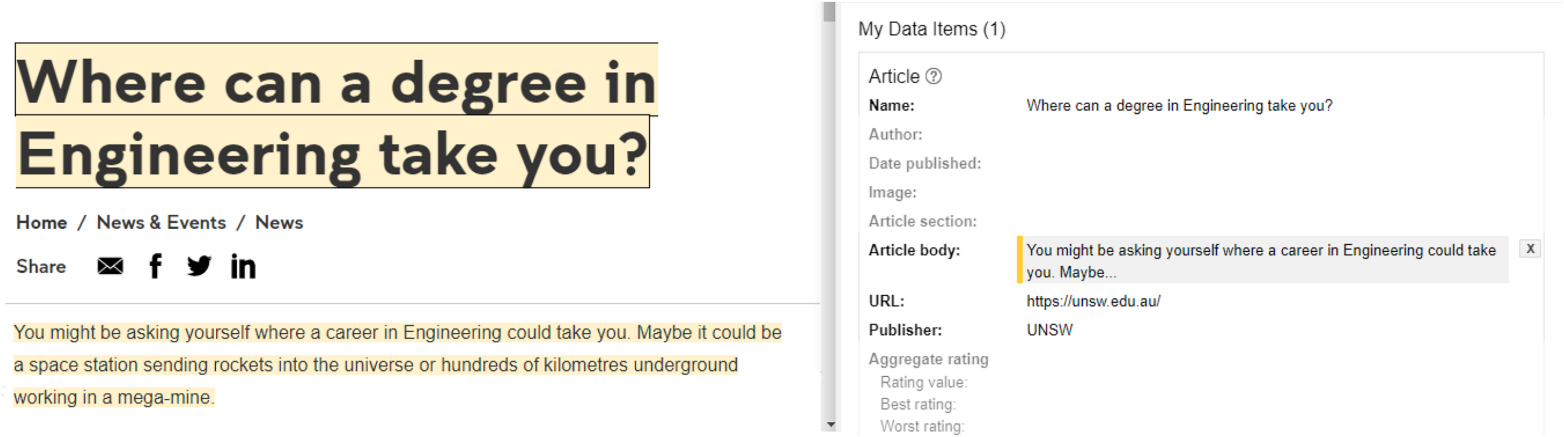
Generating JSON LD block using interactive tools.

Consider an example of a web blog, each article may have a title, domain, publishing date, and other information that may prove useful in facilitating semantic search, and therefore a webmaster must generate JSON-LD blocks with information on each article on the webpage. The service providers in the domain of search engine optimization offer interfaces to manually select and tag the relevant properties available for the article as shown in Fig. 4. In many cases, data for recommended properties may not be available or unrelated information could be present on the webpage. Therefore, the task requires cognitive skills, domain knowledge, and considerable effort for appropriate tagging.

**Figure 5:**
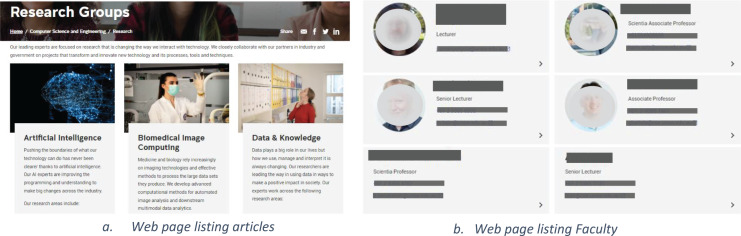
Layout and information differences in webpages.

Adding to the challenge is the fact that there is no way for the available tools to predict and adapt to the changes in the content of different web pages of the same website, as shown in Figs. 5A and 5B. There may also be differences in the information presented on a single webpage as shown in Fig. 5B where some data of some faculty members has an associated photo while for others there are no photos. Similarly, existing solutions are not capable of adapting to different sites across the web, thus creating a bottleneck for large-scale adoption of Schema.org and the semantic web.

Observing the limitations, this study finds the gaps in the adoption of practices associated with SEO and the semantic web to propose a solution for process automation thus minimizing the costs and aiding the widescale adoption of these tools. We show that, with the concentrated effort on making a small dataset of entities used in a specific domain, the proposed approach can be reliably used to identify entities automatically from web pages. Information may then be used to populate schema markup templates based on Schema.org classes. Schema markups may be integrated with web content in different formats such as microdata, JavaScript Object Notation for Linked Data (JSON-LD), and others. JSON-LD can be generated programmatically and is also recommended by the Google search engine (Developers, 2021). Using schema markups, Webmasters can make the web content more understandable to major search engines.

## Methodology

**Figure 6:**
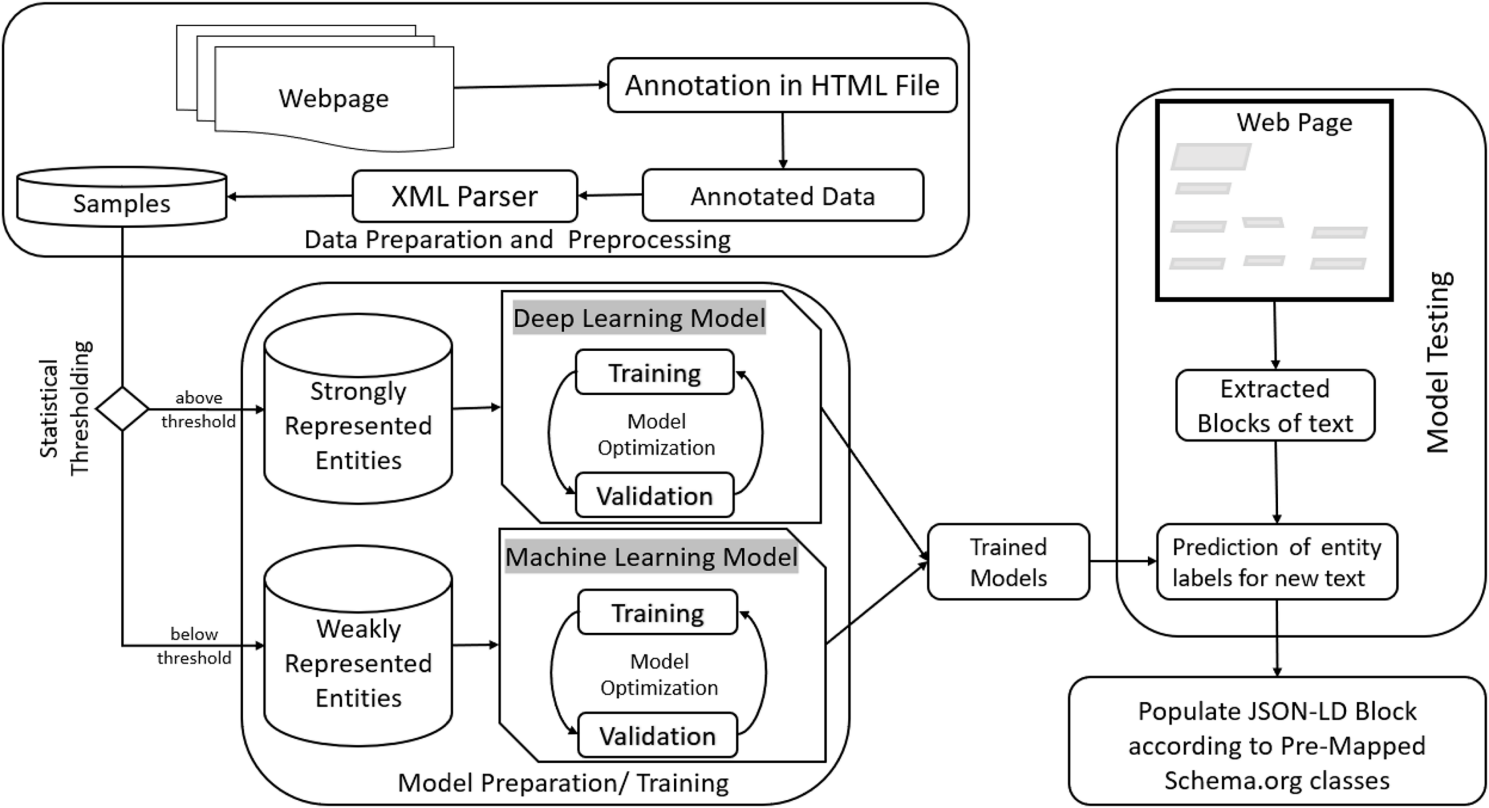
Proposed workflow diagram.

We propose our approach as a system that takes an ordinary HTML script as input and outputs the transformed HTML script after recognizing the entities and associating them with appropriate JSON-LD blocks containing Schema.org classes and properties. The system employs machine learning (ML) and deep learning (DL) models that augment the ontology given by Schema.org. The pipeline consists of preprocessing, training, and testing stages. The architecture of the proposed methodology has been given in Fig. 6.

The preprocessing stage includes steps such as annotation and parsing of data files. Samples are extracted from the annotated data and used to train machine learning and deep learning models. Trained models are used to predict entities from new data that can then be mapped to predetermined schema.org tags. The goal is achieved by extracting text blocks from raw HTML scripts and passing them through the prediction module. Once recognized as a specific entity, the information is then used to populate JSON-LD templates. Each JSON-LD block incorporates one or more entities found in the HTML content, thus transforming raw information into structured data.

A key aspect of this research is for our models to capture differences in the sequence of characters with each entity containing only a few words at most. As per the discussed characteristics of the dataset in “Data preparation and processing”, the nature of the data is such that samples of each target class are short in length and do not provide enough contextual information in form of sentences to be tackled with commonly used entity recognition techniques.

### Data preparation and processing

Web pages contain information presented differently depending on the purpose and significance of the data. To resolve the underlying challenges due to the difference in the nature of data across various domains, we narrow the scope of our work from all the web pages available on the Internet to a specific domain of academic sites. In this article, we use a subset of HTML webpages crawled for research purposes as prepared by researchers (de Freitas Veneroso & Ribeiro-Neto, 2018). The data set was chosen as it provided the raw HTML files from various academic sites. Web pages containing faculty and staff information for 12 different academic institutes were chosen arbitrarily from the data of 145 sites crawled by researchers (de Freitas Veneroso & Ribeiro-Neto, 2018). Information contained in the dataset includes a typical set of entities organized differently for each institute. In this context we are interested in the following entities: Organization, Institute, Department, Person Name, Title, Email, Contact, Address, Qualification, and Domain.

The data on web pages can be displayed in various layouts. In general, HTML tags are nested to form a hierarchy. The combination of HTML tags for a given type of web page is not standardized, therefore, extracting entities through parsing HTML DOM is not feasible, as it cannot be generalized across different websites. Thus, the dataset is prepared by annotating the HTML pages with the concepts of interest using XML tags.

Our analysis showed that the dataset had a significant imbalance in the representation of classes containing a modest number of samples for some classes compared to others. Such a limitation can happen with the web pages of other domains as well, *i.e*., different entities in a particular domain may have varying levels of representation in the webpage. To embrace such limitations, we adopt an approach whereby the problem of imbalanced data can be addressed, and the solution is capable of being adopted for any type of web page and domain.

Thus, we introduce the notion of strong and weak entities. The entities that were found frequently in the dataset are referred to as Strongly Represented Entities (SRE) whereas entities containing fewer samples are referred to as Weakly Represented Entities (WRE). The description of each entity and information about its categorization are given in Table 1.

**Table 1:** Entity description and categorization.

Entity labels	Description	Schema.org Class/Property
Strongly Represented Entities (SRE)		
Person name	Name of Person (Staff, Faculty Member) *e.g*., “Dr. John Doe”, “Doe, John”	Person:: givenName
Title	Job title, responsibility, administrative role *e.g*., “Professor”, “Dean”, “Head of Department”	Person:: jobTitle
Email	Email id *e.g*., “j.doe@tech.uni.edu”	Person:: email
Contact	Phone number/Office Phone Extension number *e.g*., “111-111-075”	Person:: telephone
Address	Office address of Faculty or Staff *e.g*., “Room7, NC Basement, New Campus”	Person:: address
URL	Website *e.g.*, “http://uni.tech.edu”	Person:: URL
Qualification	Academic qualifications *e.g.*, “Ph.D Computer Science”	Person:: hasCredential> EducationalOccupationalCredential^1^:: name
Domain	Area of expertise or specialization *e.g*., “Search Engine Optimization”	Person:: knowsAbout
**Weakly Represented Entities (WRE)**		
Organization	Name of University *e.g*., “University of Technology”	Person:: worksFor >Organization:: name
Institute	Name of teaching and training bodies (Schools, Colleges *i.e*., smaller unit/component) *e.g*., “School of Electrical and Information Systems”	Person:: worksFor >Organization >subOrganization:: name
Department	Smaller units in the Academic environment *e.g*., “Department of Computer Science”	Person >worksFor >Organization >department

**Note:**

1 EducationalOccupationalCredential is proposed to be integrated into Schema.org pending adoption from applications and websites.

Other researchers have found simpler techniques such as word replacement, word insertion, word deletion, and order manipulation to be useful for dealing with smaller datasets (Wei & Zou, 2019). Influenced by this, we adopt a simpler solution to tackle data challenges; we examine the samples in WRE and add similar samples from other academic sites.

We perform preprocessing on an annotated collection of web pages (https://github.com/B-Abbasi/Autonomous-Schema-Markup). A parsing module reads through the HTML files, extracts the annotated content, and assigns appropriate labels based on the XML tag. These annotated samples form the basis of our training and test sets. As part of the initial pre-processing steps, special characters were replaced with appropriate representations for instance “*@*” was replaced with “*[at]*”, “.” with “[dot]”, “,” with “[comma]”, “-” with “[hyphen]”, “:” with “[colon]”, “;” with “[semicolon]”. After preprocessing, data is fed to the models for the training and prediction phase described below.

### Model preparation for entity labels prediction

We use established techniques to tackle the challenge of identifying entities according to the relevant Schema.org classes. Random forest was used for model training on entities having a small number of samples in the dataset. The choice of models is driven by various factors, in the case of WREs, the limitation is the limited number of samples. To tackle this limitation, we utilize the random forest algorithm which is well known to be robust to the problem of overfitting associated with small sample size. On the other hand, we use LSTM, a deep learning algorithm for SREs, as its simpler variant, recurrent neural network suffered from the known problems of exploding gradients for long sequences of inputs (Nguyen et al., 2016). Addressing the limitation of less data for some entities while also utilizing many samples of other entities through deep learning allows for our approach to remain adaptable for new domains. The trained models can then be used to predict the entity present in the content of a web page. Once we have predicted the labels, we can use this knowledge to populate predefined JSON-LD blocks. Next, we give a general explanation of each algorithm followed by the specifics of our proposed approach.

#### Random forest for WREs

Random forest is an ensemble learning technique that uses decision trees to generate improved estimations. The tree is built in a top-down manner, using tuples in the training set. The training set gets partitioned into smaller subsets as the tree grows deeper. Information gain is calculated for each attribute for the subset of samples at each level of the tree. Information gain is based on the concept of entropy, where the entropy of a variable X is given by Eq. (1).

(1) $$H\left( X \right) = - \mathop \sum \limits_{i = 1}^n P\left( {{x_i}} \right)logP\left( {{x_i}} \right)$$

The attribute with the highest information gain is used to create branches at a given level. This partitioning minimizes the information needed to classify the tuples in the resulting subsets of samples and contains the least amount of randomness. The collection of these decision trees becomes a forest. For classification, each tree makes its prediction, and the most predicted class is chosen as the output of the forest.

A random forest can be built using bagging in addition to randomly selected features for each tree. Using a large number of trees, the random forest technique can be made robust to errors and outliers and can tackle the problem of overfitting effectively (Han, Pei & Kamber, 2011). Researchers have found the random forest to be an effective approach in the domain of Natural Language Processing (Elghazel et al., 2016).

#### Long short-term memory networks for SREs

**Figure 7:**
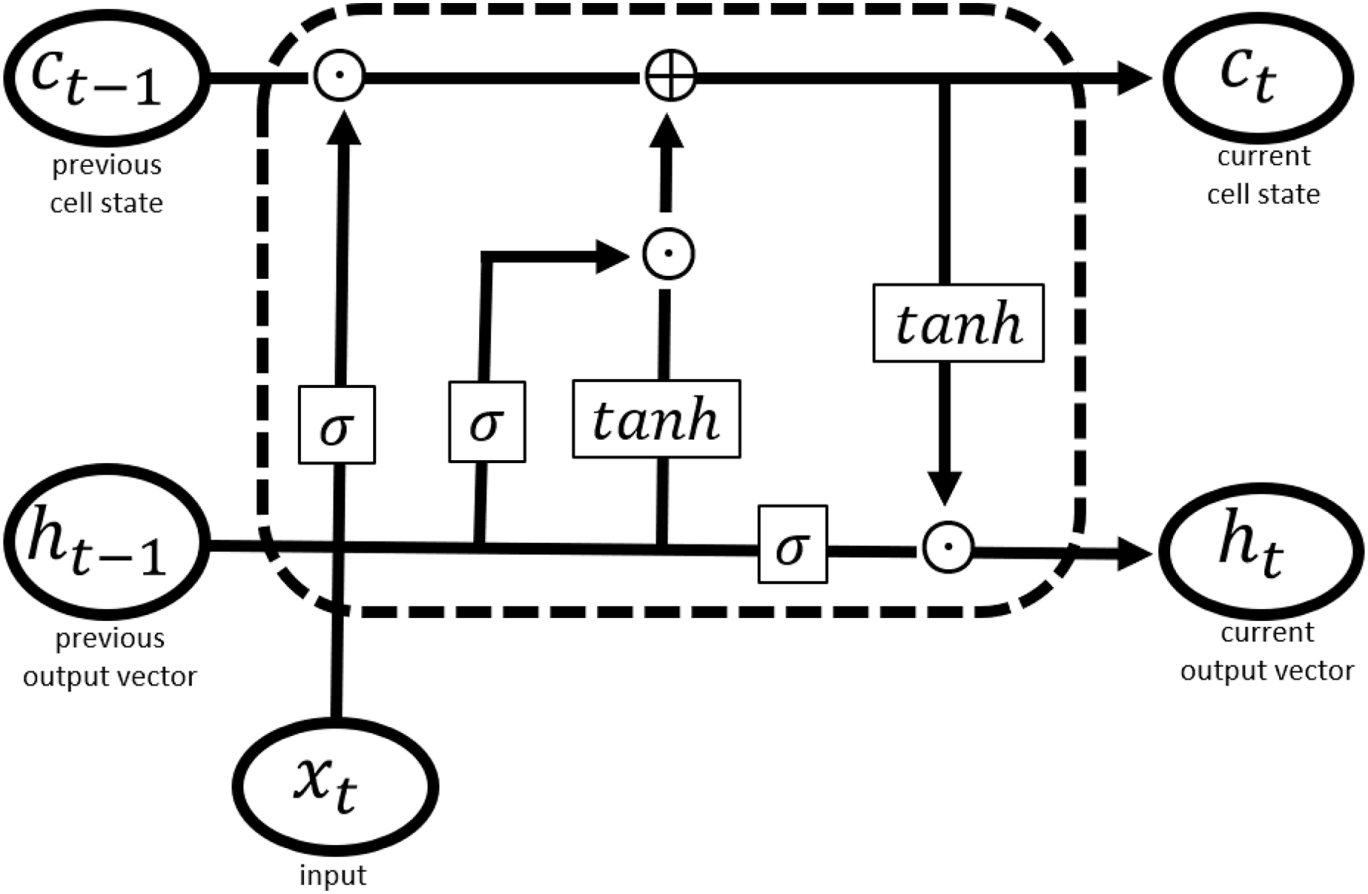
LSTM cell structure.

Long short-term memory network (LSTM) is a specialized form of recurrent neural networks (RNNs) that has been found useful where a sequence of inputs has weightage in the prediction task. Another benefit offered by LSTM over traditional neural networks is that it supports the input of variable length. LSTM and its variants have emerged as a popular choice for entity recognition tasks (Li et al., 2020). Like other deep learning approaches, it processes the training data in a complex way to enable prediction for complicated multivariate data (Graves, 2013). LSTM is designed to store and access information better than standard RNNs. It contains memory cells to control the flow of information (Graves, 2013; Hochreiter & Schmidhuber, 1997). The architecture shown in Fig. 7 allows the removal or addition of information to the cell state that is controlled through structures referred to as gates, this allows the network to track long-term dependence of input values occurring in a sequence. The sigmoid function is used to evaluate previous output and current input and to decide on how much information is to be retained in the cell state, this is referred to as the “forget gate”.

(2) $$X = \left[ {\; \matrix{ {{h_{t - 1}}} \cr {{x_t}} \cr } \; } \right]$$

(3) $${f_t} = \sigma \left( {{W_f}\cdot X + {b_f}} \right)$$

(4) $${i_t} = \sigma \left( {{W_i}\cdot X + {b_i}} \right)$$

(5) $${o_t} = \sigma \left( {{W_o}\cdot X + {b_o}} \right)$$

(6) $${c_t} = {f_t} \odot {c_{t - 1}} + {i_t} \odot tanh\; \left( {{W_c}\cdot X + {b_c}} \right)$$

(7) $${h_t} = {o_t} \odot tanh\left( {{c_t}} \right)$$

Here ${x_t}$ in Eq. (2) is the input vector, representing word embeddings. ${W_i},{W_f},\; {W_o}$are the weighted matrices and ${b_i},{b_f},\; {b_o}$ are biases of the input gate, forget gate, and output gate in Eqs. (3)–(5), respectively. $\sigma$ represents a sigmoid function and $\odot$ in Eqs. (6) and (7) represent element-wise multiplication. Eq. (7) is the final equation where ${h_t}$ is the vector representing an output, given input ${x_t}$.

The random forest and LSTM models have been trained and evaluated for their performance using standardized approaches. Accuracy and cross-entropy loss were used to evaluate the performance of the random forest and LSTM models, respectively. Details of the experiments and evaluation measures are discussed in the next section.

## Experimental evaluation and discussion

This section briefly discusses the experimental setup, evaluation measures, and results of the proposed approach. Experiments were performed on the data extracted from the annotated files. After training machine learning and deep learning models, test data was used to measure the predictive capabilities of the trained models. Finally, JSON-LD templates are populated based on the predicted classes and their associated properties found in Schema.org ontology.

### Experimental setup

We use SciKit-Learn (https://scikit-learn.org/) and PyTorch (https://pytorch.org/) to implement the standard implementations of the models used in this research. We train a random forest on the samples of WREs using information gain as the criterion for feature selection in building decision trees. In a small dataset with a weak representation of entities, we run the risk of not having sufficient vocabulary; to address this we use pre-trained 50-dimensional GloVe word embeddings based on the English Gigaword dataset (Pennington, Socher & Manning, 2014; Parker et al., 2011). English Gigaword is based on several years of newswire data from different international sources. Starting with the pre-trained vectors on this resource, our model learns task-specific embeddings to achieve the desired outcome.

LSTM was used for the prediction of SREs. We use two LSTMs together such that the output of one LSTM is passed to the second LSTM as input. The second LSTM then produces the final output. The input vector size was set to 100 whereas the size of hidden layers was set to 500. All hidden and cell states were initialized with zero and the learning rate was set to 5 × 10^−3^. During the training, process parameters are learned based on cross-entropy loss and optimized using Adam optimizer.

### Evaluation parameters

We use different performance measures to evaluate the prediction capability of each model. Accuracy was the key metric used to evaluate the models. Accuracy is calculated using the formula given in Eq. (8), where *Tp, Tn, Fp, Fn* represent *true positives, true negatives, false positives*, and *false negatives*, respectively.

(8) $$Accuracy = \; \displaystyle{{Tp + Tn} \over {Tp + Tn + Fp + Fn}}$$

Additionally, the cross-entropy loss was used to evaluate the performance of the deep learning model over multiple epochs. Loss for each sample is calculated as given in Eq. (9), here $x$ is the input, *y*_*n*_ is the actual class for n^th^ sample, C represents the number of classes.

(9) $${l_n} = \; - {w_{{y_n}}}log\displaystyle{{{\rm exp}\,\left( {{x_{n,{y_n}}}} \right)} \over {\mathop \sum \nolimits_{c = 1}^C {\rm exp}\,\left( {{x_{n,c}}} \right)}}$$

Loss aims to represent the strengths and weaknesses of a trained model with a single number. During the training process, we aim to minimize the value of loss to reach an optimal model that generalizes well for the unseen data, thus we are also able to check for scenarios such as overfitting and underfitting. Cross-entropy loss is particularly useful in the case of an unbalanced dataset.

### Random forest performance

Random forest was used for training a model to predict entities belonging to WRE. First, the dataset is split into a training set and a test (holdout) set. We validate the performance of the trained model through cross-validation as well as holdout validation. A training set is used to train and validate the model prediction capabilities through 10-fold cross-validation. The average accuracy of the model in cross-validation was 0.98. The trained model was also evaluated on the holdout set to verify its performance on unseen data where the model had an accuracy of 0.94, as shown in Table 2.

**Table 2:** Prediction score for weakly represented entities.

Model	Evaluation criteria	Accuracy
WRE—random forest	10-Fold cross-validation	0.98
	Hold-out validation	0.94

Misclassification of samples can be explained by the nature of these entities as the samples may not be highly distinctive for the chosen domain of an academic website. The test set consisted of 20 samples representing the entity Department. Out of these 18 were correctly classified, whereas two false negatives were classified as Institute. All samples of the entity Institute were correctly predicted, however, the accuracy for this class was affected due to the false positives. Table 3 shows a confusion matrix for the prediction results of the random forest algorithm, showing the actual and predicted values for the WREs. The first column provides information on the actual class of samples whereas the first row represents predicted classes for the samples.

**Table 3:** Weakly represented entities (WRE) multi-class confusion matrix.

	Organization	Department	Institute
Organization	6	0	0
Department	0	18	2
Institute	0	0	27

### LSTM performance on test set

We use accuracy as the final measure of the performance of the LSTM model to predict SREs. Loss and accuracy improved with more training. After each training cycle, the resulting model was evaluated on the test set. While the accuracy of some epochs was higher, we report the average value to give a general idea of the predictive capability of our model. The average accuracy on the test set over multiple training cycles was recorded at 0.94. Training and test losses recorded for individual epochs are given in Table 4. Training loss decreased from 0.7 in the first epoch to 0.08 in the eighth epoch. Results of training are confirmed by monitoring test loss for each epoch, which decreased from 0.5 in the first epoch to 0.2 in the tenth epoch, correspondingly accuracy of the model also improved.

**Table 4:** Loss & accuracy for training & test set for strongly represented entities (SRE).

Epoch #	Training loss	Test loss	Test accuracy
1	0.789	0.506	0.86
2	0.242	0.271	0.93
3	0.170	0.276	0.94
4	0.112	0.287	0.95
5	0.117	0.205	0.96
6	0.134	0.325	0.95
7	0.103	0.322	0.95
8	0.087	0.365	0.96
9	0.090	0.399	0.94
10	0.107	0.265	0.96

**Figure 8:**
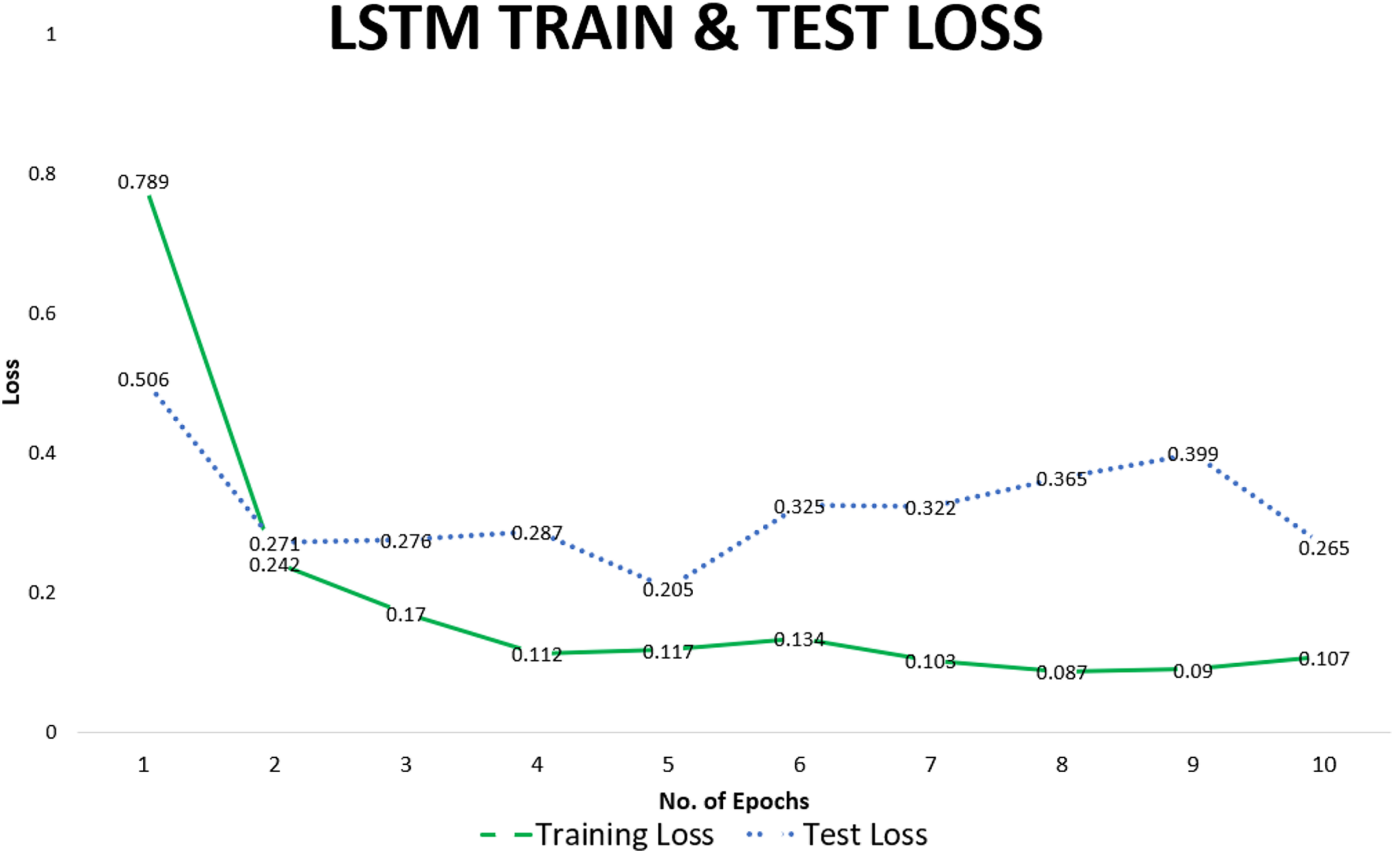
LSTM-train & test loss.

The training loss declined rapidly after the first epoch and varied in a narrow range over subsequent epochs, whereas test loss remained stable indicating that the model generalizes well to be able to predict unseen data. This can be observed from the visual representation of the data points given in Fig. 8.

Results were further analyzed using a confusion matrix. False positives in the case of Address were commonly mistaken as a person’s name which may be attributed to the fact that buildings in academic institutes are often named after famous scientists of a specific domain. Similarly, several samples from other entities were predicted as Person Name, higher number of false positives from a specific class indicates a need for larger training data and if not possible at least more training samples from the two classes that model is unable to distinguish, especially for the entities where large variation in values is observed. Entity “Person Name” had the largest representation in the test set, TP of 348 out of 365 was achieved by the model. Entity “Domain” had the smallest representation in the test set, TP of 38 out of 65 was achieved. By increasing the representation of this class in the training set, false negatives can be minimized. Table 5 shows the multi-class confusion matrix for the LSTM model on the test set, where the first column lists the actual class of the samples, and first row indicates the predicted class for respective samples. Along the diagonal are the true positives whereas false negatives and false positives are shown in respective rows and columns.

**Table 5:** Strongly represented entities (SRE) multi-class confusion matrix.

	Address	Person name	Email	Contact	Title	Domain	Qualification
Address	153	27	0	0	0	1	9
Person name	0	348	0	1	0	1	15
Email	0	1	210	1	0	0	2
Contact	0	1	0	184	0	0	1
Title	3	22	0	0	290	0	13
Domain	1	16	0	2	6	38	2
Qualification	0	0	0	0	0	0	141

**Figure 9:**
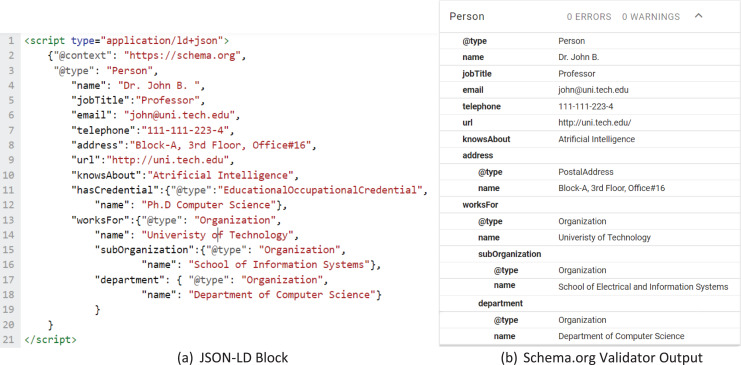
JSON-LD block to be embedded in webpage.

Creation of a JSON-LD block after the prediction of the appropriate entity label is achieved using a predefined JSON-LD template that utilizes mapping of entity labels with associated Schema.org classes and properties. As discussed, Schema.org provides guidelines on the use of properties of different classes and defines how relationships among different classes can be mapped. A web page containing information about a person can have a JSON-LD block containing name, job title, email, and works-for properties. While some properties simply hold text values others are mapped to classes, for example, the “WorksFor” property holds an object of the “Organization” class. This means that the person works for an organization, where the organization may be described through several properties in Schema.org ontology depending on the availability of the data. An example of a JSON-LD block containing the classes and properties used in this research is shown in Fig. 9A. Different combinations are possible for classes and properties depending upon the availability of data; and all of them must follow the structure and rules defined by the ontology, for this reason, schema validators have been introduced. This helps SEO experts to confirm the correctness of the JSON-LD block. We used the JSON-LD validator (https://validator.schema.org/) provided by Schema.org to validate this populated block, Fig. 9B shows the results of the validator.

To the best of the authors' knowledge, the proposed work is novel in its objectives. Recently researchers have started focusing on information extraction from websites, however, most do not tackle such data due to its inherent challenges and limitation. In a recent study, Neumann, Linder & Desmarais (2022) note that manual methods are used for the extraction of useful information from government websites, thus proposing an automated method of data extraction. While their proposed methodology supports multiple file formats commonly found on such websites, the module for information extraction from HTML files mainly depends on the application of previous work by Kohlschutter, Fankhauser & Nejdl (2010), In their experiments, the focus remains on sentence-based content such as blogs and articles. Similarly, Feng et al. (2022) proposed a two-stage learning technique for named entity recognition. Their approach focuses on the use of deep learning techniques for sentences containing terms specific to a specific domain. This limits the application of these techniques for the scenario discussed in current research and renders them unsuitable in the context of the semantic web.

## Conclusion

This study aimed to facilitate content discovery in the semantic web, utilizing the ontology structure described by Schema.org and SEO practices. The authors proposed and implemented a system that utilizes deep learning and machine learning techniques to predict appropriate entities for given text sequences. Based on the analysis of data, it was observed that a few classes were severely under-represented. Therefore, two different approaches were proposed to handle entity prediction. The long-short term memory (LSTM) network for strongly represented entities (SRE) and random forests for prediction of weakly represented entities (WRE). The effectiveness of the proposed system is validated by the accuracy of 0.94 by the random forest model and 0.94 by the LSTM model on a sample dataset. The consistent performance of the system with a relatively small number of training samples indicates the effectiveness of the proposed approach, thus reducing the required labeling workload in practical applications.

The results of experiments show that various entities from unstructured or semi-structured web content can be automatically mapped to the classes and properties of schema.org. The proposed architecture may be adopted for web pages of other domains as well and the trained models may be integrated easily with existing web services. The current study does not explore system-based recommendations for schema.org classes and properties. Future works may also focus on automated recommendations of schema.org classes and properties given a specific text. Search engines have adopted varying standards on the Schema.org properties as mandatory or optional while populating JSON-LD scripts. Such differences and their impact may also be studied in future research.

## Additional Information and Declarations

### Data Availability

The following information was supplied regarding data availability: The code and data are available at Zenodo: Burhan Abbasi. (2022). B-Abbasi/Autonomous-Schema-Markup: Research Publication 2022 (v0.5). Zenodo. https://doi.org/10.5281/zenodo.7234702.

## References

[ref-1] Akram M, Sohail I, Hayat S, Shafi MI, Saeed U (2010). Search engine optimization techniques practiced in organizations: a study of four organizations. Journal of Computing.

[ref-2] Ankolekar A, Krötzsch M, Tran TT, Vrandecic D (2007). The two cultures: mashing up Web 2.0 and the semantic web.

[ref-12] Ansari B, Barati M, Martin EG (2022). Enhancing the usability and usefulness of open government data: A comprehensive review of the state of open government data visualization research. Government Information Quarterly.

[ref-3] Anthes G (2012). HTML5 leads a web revolution. Communications of the ACM.

[ref-4] Aryshandy G, Fernando Y, Dharmaputra RT, Ikhsan RB, Wahyuni-TD IS (2021). How does search engine optimization affect outcomes of electronic marketing strategy?.

[ref-5] Asgari-Chenaghlu M, Feizi-Derakhshi MR, Farzinvash L, Balafar M, Motamed C (2022). CWI: a multimodal deep learning approach for named entity recognition from social media using character, word and image features. Neural Computing and Applications.

[ref-6] Baye MR, Santos BDL, Wildenbeest MR (2016). Search engine optimization: what drives organic traffic to retail sites?. Journal of Economics & Management Strategy.

[ref-7] Berners-Lee T, Hendler J, Lassila O (2001). The semantic web. Scientific American.

[ref-8] Bevendorff J, Stein B, Hagen M, Potthast M (2018). Elastic chatnoir: search engine for the clueweb and the common crawl.

[ref-9] Bhandari RS, Bansal A (2018). Impact of search engine optimization as a marketing tool. Jindal Journal of Business Research.

[ref-10] Bhardwaj B, Ahmed SI, Jaiharie J, Dadhich RS, Ganesan M (2021). Web scraping using summarization and named entity recognition (NER).

[ref-11] Bhatia P (2019). Data mining and data warehousing: principles and practical techniques.

[ref-13] Chilet JA, Chen C, Lin Y (2016). Analyzing social media marketing in the high-end fashion industry using Named Entity Recognition.

[ref-15] Chou C-L, Chang C-H (2017). Mining features for web ner model construction based on distant learning.

[ref-16] Chou C-L, Chang C-H, Lin Y-H, Chien K-C (2020). On the construction of web NER model training tool based. ACM Transactions on Asian and Low-Resource Language Information Processing (TALLIP).

[ref-17] De Freitas Veneroso JM, Ribeiro-Neto B (2018). Entity name extraction from faculty directories.

[ref-22] Developers G (2021). Understand how structured data markup works. Google Search Central.

[ref-18] Dye K (2008). Website abuse for search engine optimisation. Network Security.

[ref-19] Elghazel H, Aussem A, Gharroudi O, Saadaoui W (2016). Ensemble multi-label text categorization based on rotation forest and latent semantic indexing. Expert Systems with Applications.

[ref-20] Feng X, Li Y, Hang Z, Fan Z, Yu Q, Xin R (2022). TBR-NER: research on COVID-19 text information extraction based on joint learning of topic recognition and named entity recognition. Journal of Sensors.

[ref-21] Fu J, Huang X, Liu P (2021). SpanNER: named entity Re-/Recognition as span prediction. Proceedings of the 59th Annual Meeting of the Association for Computational Linguistics and the 11th International Joint Conference on Natural Language Processing.

[ref-23] Graves A (2013). Generating sequences with recurrent neural networks. ArXiv preprint.

[ref-24] Griazev K, Ramanauskaitė S (2021). Multi-purpose dataset of webpages and its content blocks: design and structure validation. Applied Sciences.

[ref-25] Gudivada VN, Rao D, Paris J (2015). Understanding search-engine optimization. Computer.

[ref-26] Guha RV, Brickley D, Macbeth S (2016). Schema. org: evolution of structured data on the web. Communications of the ACM.

[ref-27] Guo X, Lu S, Tang Z, Bai Z, Diao L, Zhou H, Li L (2022). CG-ANER: enhanced contextual embeddings and glyph features-based agricultural named entity recognitio. Computers and Electronics in Agriculture.

[ref-28] Han J, Pei J, Kamber M (2011). Data mining: concepts and techniques.

[ref-29] Hitzler P (2021). A review of the semantic web field. Communications of the ACM.

[ref-30] Hochreiter S, Schmidhuber J (1997). Long short-term memory. Neural Computation.

[ref-31] Izo F, Oliveira E, Badue C (2022). Named entities as a metadata resource for indexing and searching information.

[ref-32] Jing G, Liu L, Wang Z, Zhang Y, Qian L, Gao C, Liu X (2021). Microbiome search engine 2: a platform for taxonomic and functional search of global microbiomes on the whole-microbiome level. Msystems.

[ref-33] Kellogg G, Champin P-A, Longley D (2020). JSON-LD 1.1. https://www.w3.org/TR/2020/REC-json-ld11-20200716/.

[ref-34] Khadir AC, Aliane H, Guessoum A (2021). Ontology learning: grand tour and challenges. Computer Science Review.

[ref-35] Khattak FK, Jeblee S, Pou-Prom C, Abdalla M, Meaney C, Rudzicz F (2019). A survey of word embeddings for clinical text. Journal of Biomedical Informatics.

[ref-36] Kim DJ, Hebeler J, Yoon V, Davis F (2018). Exploring determinants of semantic web technology adoption from IT professionals’ perspective: industry competition, organization innovativeness, and data management capability. Computers in Human Behavior.

[ref-37] Kohlschutter C, Fankhauser P, Nejdl W (2010). Boilerplate detection using shallow text features.

[ref-38] Leitner E, Rehm G, Moreno-Schneider J (2019). Fine-grained named entity recognition in legal documents.

[ref-39] Lewandowski D, Sunkler S, Yagci N (2021). The influence of search engine optimization on google’s results: a multi-dimensional approach for detecting SEO.

[ref-40] Li J, Sun A, Han J, Li C (2020). A survey on deep learning for named entity recognition. IEEE Transactions on Knowledge and Data Engineering.

[ref-41] Manning C, Schutze H (1999). Foundations of statistical natural language processing.

[ref-42] Moon S, Neves L, Carvalho V (2018). Multimodal Named Entity Recognition for Short Social Media Posts. Proceedings of the 2018 Conference of the North American Chapter of the Association for Computational Linguistics: Human Language Technologies.

[ref-43] Nadeau D, Sekine S (2007). A survey of named entity recognition and classification. Lingvisticæ Investigationes.

[ref-44] Neumann M, Linder F, Desmarais B (2022). Government websites as data: a methodological pipeline with application to the websites of municipalities in the United States. Journal of Information Technology & Politics.

[ref-45] Nguyen T, Nguyen D, Rao P (2020). Adaptive name entity recognition under highly unbalanced data. ArXiv preprint.

[ref-46] Nguyen TH, Sil A, Dinu G, Florian R (2016). Toward mention detection robustness with recurrent neural networks. ArXiv preprint.

[ref-47] Nie Y, Tian Y, Wan X, Song Y, Dai B (2020). Named entity recognition for social media texts with semantic augmentation.

[ref-48] Parker R, Graff D, Kong J, Chen K, Maeda K (2011). English gigaword fifth edition LDC2011T07.

[ref-49] Patel-Schneider PF (2014). Analyzing schema.org. The Semantic Web–ISWC 2014.

[ref-50] Pennington J, Socher R, Manning CD (2014). GloVe: global vectors for word representation. Proceedings of the 2014 Conference on Empirical Methods in Natural Language Processing (EMNLP).

[ref-51] Saeeda L, Med M, Ledvinka M, Blasko M, Kremen P (2020). Entity linking and lexico-semantic patterns for ontology learning.

[ref-52] Şah M, Hall W, Gibbins NM, Roure DCD (2007). Semport: a personalized semantic portal.

[ref-53] Saini DKJB, Patil P, Gupta KD, Kumar S, Singh P, Diwakar M (2022). Optimized web searching using inverted indexing technique.

[ref-54] Schultheiß S, Lewandowski D (2020). Outside the industry, nobody knows what we do SEO as seen by search engine optimizers and content providers. Journal of Documentation.

[ref-55] Sporny M, Kellogg G, Lanthaler M (2014). JSON-LD 1.0: a JSON-based serialization for linked data. http://www.w3.org/TR/2014/REC-json-ld-20140116/.

[ref-56] Stolz A, Hepp M, Hemminger A (2017). Representing fashion product data with schema.org: approach and use cases.

[ref-57] Vanessa L, Victoria U, Marta S, Enrico M (2011). Is question answering fit for the semantic web?: a survey. Semantic Web.

[ref-58] Wang S, Zhou W, Jiang C (2020). A survey of word embeddings based on deep learning. Computing.

[ref-59] Wei J, Zou K (2019). Eda: easy data augmentation techniques for boosting performance on text classification tasks. Proceedings of the 2019 Conference on Empirical Methods in Natural Language Processing and the 9th International Joint Conference on Natural Language Processing (EMNLP-IJCNLP).

[ref-60] Yadav V, Bethard S (2019). A survey on recent advances in named entity recognition from deep. CoRR.

[ref-61] Zhang X, Zhan Z, Holtz M, Smith AM (2018). Crawling, indexing, and retrieving moments in videogames.

